# Nuclear Magnetic Resonance Spectroscopy in Clinical Metabolomics and Personalized Medicine: Current Challenges and Perspectives

**DOI:** 10.3389/fmolb.2021.698337

**Published:** 2021-09-20

**Authors:** Marine P. M. Letertre, Patrick Giraudeau, Pascal de Tullio

**Affiliations:** ^1^ Université de Nantes, CNRS, CEISAM UMR 6230, Nantes, France; ^2^ Metabolomics Group, Center for Interdisciplinary Research of Medicine (CIRM), Department of Pharmacy, Université de Liège, Liège, Belgique

**Keywords:** nuclear magnetic resonance, clinical metabolomics, personalized medicine, spectroscopy, biomarkers

## Abstract

Personalized medicine is probably the most promising area being developed in modern medicine. This approach attempts to optimize the therapies and the patient care based on the individual patient characteristics. Its success highly depends on the way the characterization of the disease and its evolution, the patient’s classification, its follow-up and the treatment could be optimized. Thus, personalized medicine must combine innovative tools to measure, integrate and model data. Towards this goal, clinical metabolomics appears as ideally suited to obtain relevant information. Indeed, the metabolomics signature brings crucial insight to stratify patients according to their responses to a pathology and/or a treatment, to provide prognostic and diagnostic biomarkers, and to improve therapeutic outcomes. However, the translation of metabolomics from laboratory studies to clinical practice remains a subsequent challenge. Nuclear magnetic resonance spectroscopy (NMR) and mass spectrometry (MS) are the two key platforms for the measurement of the metabolome. NMR has several advantages and features that are essential in clinical metabolomics. Indeed, NMR spectroscopy is inherently very robust, reproducible, unbiased, quantitative, informative at the structural molecular level, requires little sample preparation and reduced data processing. NMR is also well adapted to the measurement of large cohorts, to multi-sites and to longitudinal studies. This review focus on the potential of NMR in the context of clinical metabolomics and personalized medicine. Starting with the current status of NMR-based metabolomics at the clinical level and highlighting its strengths, weaknesses and challenges, this article also explores how, far from the initial “opposition” or “competition”, NMR and MS have been integrated and have demonstrated a great complementarity, in terms of sample classification and biomarker identification. Finally, a perspective discussion provides insight into the current methodological developments that could significantly raise NMR as a more resolutive, sensitive and accessible tool for clinical applications and point-of-care diagnosis. Thanks to these advances, NMR has a strong potential to join the other analytical tools currently used in clinical settings.

## Clinical Metabolomics and Personalized Medicine

**FIGURE 1:**
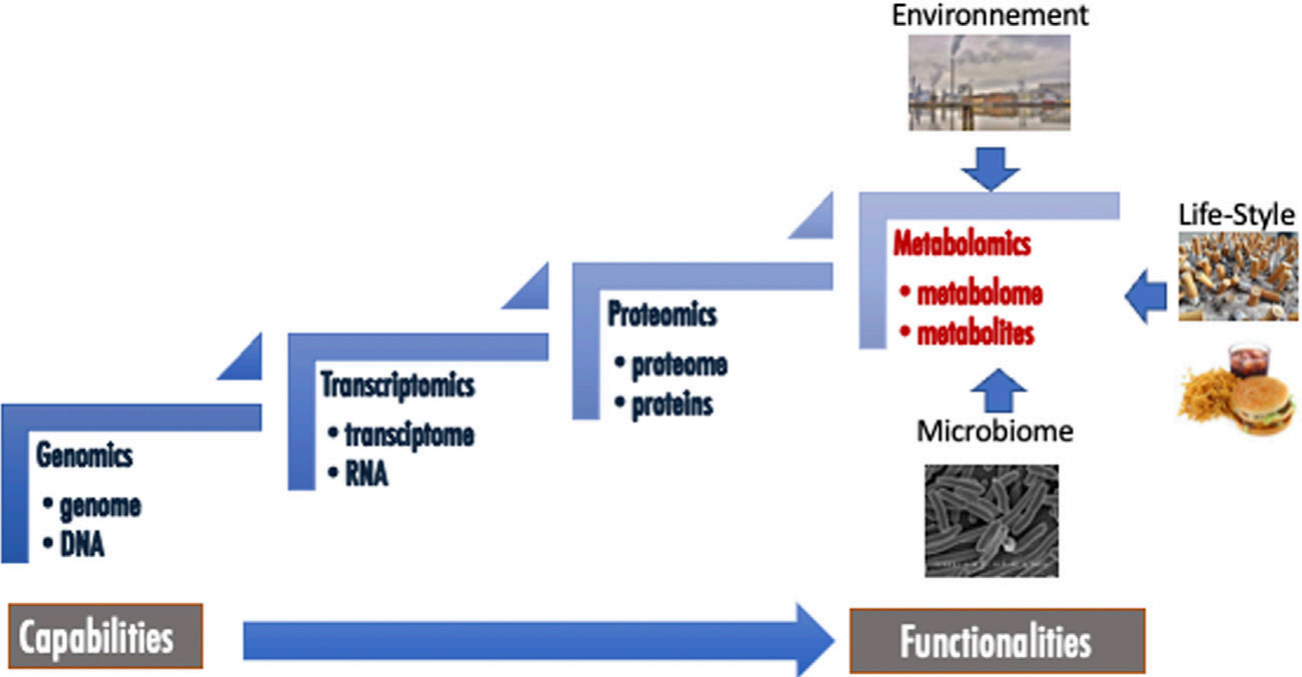
Metabolomics in the field of other omics.

Amongst “omics” approaches, metabolomics is generally presented as the last that appeared in terms of occurrence and development, but also as the final biological and biochemical stones in the complex networks of organisms. Indeed, this approach aims at identify and quantify organic low molecular weight molecules (<1,500 Da) belonging to different classes of metabolites (Nicholson and Lindon, 2008; Patti et al., 2012). These metabolites form the metabolome, which is composed of endogenous but also exogenous biochemicals coming from environment, life-style, food, medicines, microbiome and which could be involved in catabolic and anabolic reactions (Figure 1). Metabolomics is clearly correlated with the functionality of the organism, while the other “omics” such as genomics, transcriptomics and proteomics, are closest to its capabilities (Figure 1). While the applications of metabolomics are numerous and varied in areas such as food and natural products quality controls (Lee et al., 2017; Li et al., 2021), environmental studies (Viant, 2009; Bedia et al., 2018) or agriculture (Kumar et al., 2017), the most highlighted and probably the most promising application fields of this methodology are clinical metabolomics and personalized medicine (Wishart, 2016; Li B. et al., 2017; Kohler et al., 2017; Nielsen, 2017; Tolstikov et al., 2017; Trivedi et al., 2017; Jacob et al., 2019; Pang et al., 2019). Clinical metabolomics is a general terminology that deals with all the applications of this approach that involve human subjects. It includes fundamental studies of diseases (Nielsen, 2017), searches for new biomarkers discovery (Kohler et al., 2017) as well as for new therapeutic targets and drug development processes (Powers, 2014; Cuperlovic-Culf and Culf, 2016; Frédérich et al., 2016), epidemiology (Moayyeri et al., 2013; Chan et al., 2017; Yu et al., 2019) and, recently, appears as an interesting tool in the development of personalized or precision medicine (Wishart, 2016; Li B. et al., 2017; Trivedi et al., 2017; Jacob et al., 2019). Indeed, medical care is continuously evolving toward a more patient-centered approach.

Personalized medicine is probably the most important paradigm change in medical care that occurred during the last few years and is clearly the future of modern medicine (Di Sanzo et al., 2017; Elemento, 2020). This approach attempts to optimize the therapies and the patient care based on the individual patient characteristics (*i.e.*, genetic dispositions, phenotype, life-style, environmental parameters … ) and is expected to improve treatment efficacy and the quality of life of the patients. The keystone of this approach is linked to the ability of the clinicians 1) to precisely characterize the disease, 2) to stratify the patients (*i.e.*, according to genotype but also to phenotype), 3) to select the right treatments adapted to the disease and the patient conditions and 4) to follow the pathology and the treatment outcomes and to predict their evolutions. Thus, the classical clinical tools currently used must be improve so personalized medicine can reach such ambitious objectives. Modern and innovative approaches are instrumental to propose more robust and trustworthy preventive/prognostic solutions, in order to measure, integrate and model informative data that could help clinicians to select the best option for the patient care. In this way, biomarker discovery and measurement for longitudinal patient follow-up appear as the cornerstones of personalized medicine (Kohler et al., 2017)

Given all these demands and needs, it is clear that clinical metabolomics should play an important role in changing the way patient care is approached. Patients’ metabolic profiles are very dynamic and can be influenced by external or internal stimuli, lifestyle and clinical changes and can be used to monitor and explore cellular or tissue homeostasis as well as physiological and pathological conditions (Wishart, 2016; Tolstikov et al., 2017). A robust, quantitative and reproducible study of their metabolome is then essential to accurately define their phenotype (Jacob et al., 2019). Moreover, human pathologies are often complex, with multiple molecular pathogenesis and heterogeneous clinical pictures between patients and are not only driven by genetics but can also be strongly influenced by the function or dysfunction of different metabolic and biochemical networks. These networks are highly controlled by several internal but also external patient parameters, and therefore, the identification and integration of these parameters is essential for a better understanding of the mechanisms that led to the causality and the development of a pathology. Since it allows measuring the occurrence and variations of metabolites in organs, tissues and biofluids to be reported in a spatial and temporal manner, clinical metabolomics is to be an essential tool and will play a major role for the search for biomarkers, the identification of biochemical pathways involved in a pathology, the study of the environment and lifestyle influences and the treatment follow-up. However, there are still many obstacles and challenges to overcome in order to bring this approach from the laboratory to the clinical practice (Pinu et al., 2019).

The study of the metabolome and the monitoring of metabolites can be considered through two approaches: non-targeted approach and targeted approach. Non-targeted (or untargeted) metabolomics could be defined as the comprehensive and extensive measurement of a larger number of metabolites without a selection based on chemical class or biological activity. This approach is most commonly used for without *a priori* exploratory studies of pathologies and for the discovery of new specific biomarkers. Targeted or biology-driven metabolomics is the analysis of selected, chemically similar or groups of biochemically annotated metabolites such as known clinical biomarkers. It deals with quantitation or semi-quantitation of a set of known metabolites. It currently requires prior knowledge of the chemical or spectral properties of the metabolites of interest. This approach is used to study particular pathways, chemical families or biological activities and is mandatory for validating the metabolites identified by an untargeted strategy. Targeted metabolomics is particularly suited to the longitudinal studies and monitoring of patients and treatments that are essential in personalized medicine. Some classes of metabolites have led to the development of specific metabolomics fields. Lipids, which are considered an essential and crucial class of compounds, led to an “omics” approach named lipidomics, while sugars are studied in glycomics. Going one step further, fluxomics, which is the analysis of metabolic fluxes relying on labeled metabolic precursors, represents a very interesting approach for the in-depth study of the intracellular metabolism as well as the biochemical and metabolic pathways of an organism.

**FIGURE 2:**
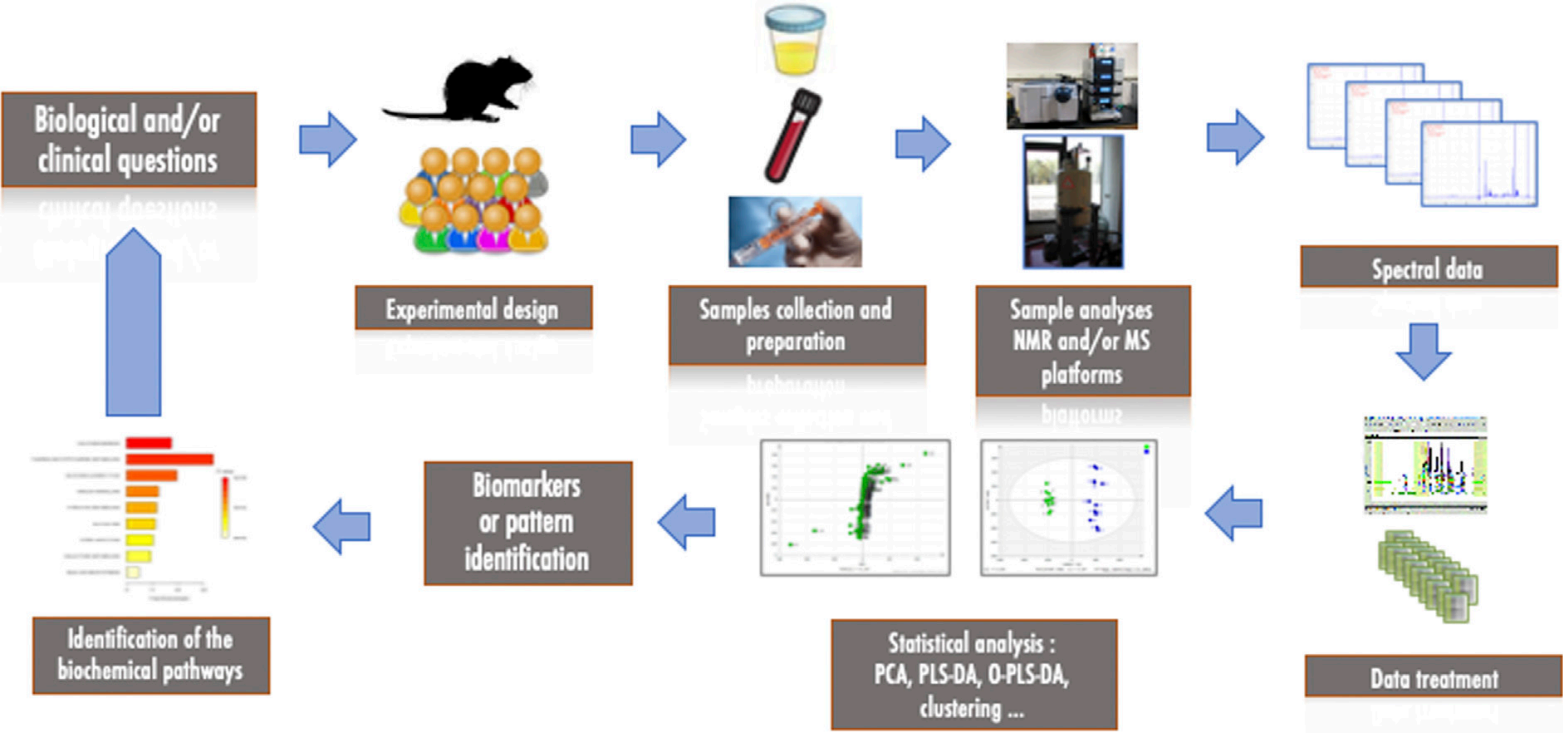
Workflow of a metabolomics study.

The classical workflow of a metabolomics study consists of several steps, as shown in Figure 2. The first step starts with the biological and/or clinical questions and leads to the experimental design, the choice of models and samples to be collected and analyzed (biofluids, biopsies, cells). The second important step is the measurement and analysis of the collected samples using high-throughput technological platforms. After the measurement and pre-processing of the data, statistical analyses are necessary to extract the most relevant information to interpret the results biologically and to identify metabolites or patterns that could be considered as biomarkers of the pathology of interest. Depending on the structure of the data, this usually requires reducing the size of the data via multivariate statistical analysis or applying classical univariate approaches. As these analyses deal with variance, all experimental and analytical variabilities must be minimized in order to reduce noise, avoid confounding factors and maximize response. Finally, the features that have been identified can be correlated to biochemical pathways and interpreted in the light of the original question and/or hypothesis. Metabolomics is thus a highly collaborative field that requires interaction between biologists, analytical chemists and statisticians.

The accurate and complete measurement of the metabolome is not an easy task at the analytical level (Kohler et al., 2017). Indeed, the great diversity of metabolites both at the physico-chemical level, the broad range of concentrations (up to mmol/L at best for the most concentrated metabolites) as well as the associated dynamic range detection issues represent probably the most important and specific challenges for the classically used analytical methods. Moreover, the analysis of complex biological matrices is further hampered by the presence of proteins, high ionic strength and the sample heterogeneity. Therefore, pre-treatment of the samples is often necessary to reduce these problems and to adapt the samples to the analytical method. However, it is obvious that the more complex and time-consuming this treatment is, the higher the risk of altering the sample (in terms of metabolites composition) and the higher the risk of introducing experimental variability.

Although the first work identified as relating to metabolomics involved GC-MS in the 70th (Zlatkis and Liebich, 1971), Nuclear Magnetic Resonance (NMR) quickly appeared to be a powerful analytical technique in this field. This is mainly due to the fact that NMR is a highly robust, reproducible and non-destructive method that can be straightforwardly adapted to the analysis of complex media (Emwas et al., 2019; Wishart, 2019; Sahoo et al., 2020). Largely in minority before the 2000s, the use of mass spectrometry (MS), coupled with chromatographic techniques (Gas or liquid chromatographies- GC or LC) for metabolome analysis has also been developed (Gowda and Djukovic, 2014; Beale et al., 2018; Cui et al., 2018; Rampler et al., 2021). Indeed, the need to better understand and characterize the metabolome, as well as the advent of concepts such as biological systems and metabolic networks and the use of metabolomics in the discovery of disease-specific biomarkers, have made it necessary to increase the number of metabolites identified, especially those present in lower concentrations. In this context, mass spectrometry coupled with chromatographic techniques naturally appeared to be the most suitable analytical platform, thanks to its favorable limit of detection. This is mainly due to the rapid technological progress of this technique, the accessible cost of routine instrumentation as well as its impressive sensitivity compared to NMR. This have progressively reversed the situation to the point where at present the use of MS is applied in the majority of metabolomics studies. This technological evolution inevitably raises the question of the future of NMR in metabolomics and more particularly in clinical metabolomics. On the one hand, if one looks at the basic opposition of the two techniques by comparing their sensitivity and the number of metabolites detected, it’s a done deal. On the other hand, if we look in more details at the situation, at the real needs in clinical metabolomics, and if we humbly remember that no single analytical technique can answer 100% of the questions, nor cover 100% of the needs, it is possible to consider these two approaches as perfectly complementary and equally essential, each with its advantages, covering the deficiencies of the other (this complementarity will be discussed in detail later in the article). The right tool(s) for the right biological question(s) must be the rule. Besides NMR and LC-MS (and to a lesser extent GC-MS), which are the two most widespread platforms, other approaches have also been or are being explored, such as vibrational spectroscopy (*i.e.*, FT-IR and Raman) (Du et al., 2020; Sherman et al., 2020) and capillary electrophoresis coupled or not with a MS detector (Maier and Schmitt-Kopplin, 2016; Sasaki et al., 2019).

There are many studies comparing the advantages and disadvantages of NMR and MS in the field of metabolomics and it is not our intention here to add one more (Frédérich et al., 2016; Kohler et al., 2016; Emwas et al., 2019; Wishart, 2019). Instead, we want to focus on what NMR can bring to clinical metabolomics and personalized medicine, how it can address the challenges of these fields and how its use provides them with a new opportunity and an added value (Markley et al., 2017; Emwas et al., 2019; Takis et al., 2019; Giraudeau, 2020). Thus, the three following chapters respectively highlight the current position of NMR in clinical metabolomics, the complementarity of NMR with MS and the recent and future developments of NMR in the same field.

## Nuclear Magnetic Resonance in Clinical Metabolomics and Personalized Medicine

To fully understand the role that NMR can play in clinical metabolomics and personalized medicine, it is important to keep in mind the weaknesses and strengths of this technique in these particular applications. With this in mind, we will examine how this analytical approach has been used advantageously and successfully in a wide range of research and projects and how its weaknesses can be improved.

### Initial Limitations of Nuclear Magnetic Resonance Approach

When discussing NMR and comparing it to other analytical techniques, particularly MS, its lack of sensitivity and resolution are often highlighted. In fact, despite significant technical improvements in recent years, the limit of detectable and quantifiable concentrations for hydrophobic metabolites in NMR is often in the micromolar range, - a few tens of micromolar at best on the typical metabolomics platforms. Moreover, the absence of separative techniques preceding the NMR analysis often leads to the overlapping of certain signals, which sometimes drastically reduces the resolution, especially in 1D-NMR. While multi-dimensional NMR techniques greatly improve the resolution (see *Recent and Future Developments in Nuclear Magnetic Resonance-Based Metabolomics*), sensitivity remains the main weak point of NMR. Knowing that many metabolites in biofluids have concentrations often close to or below the detection limit of NMR, it is obvious that this technique can only visualize a small part of the metabolome. However, this limitation should be counterbalanced, on the one hand, because the part of the metabolome that can be visualized and quantified by NMR is often of crucial importance, and on the other hand because reliable metabolomics analysis can be multiple and should not be boiled down to the detection of a maximum number of metabolites.

### Advantages and Specificities of the Nuclear Magnetic Resonance Approach and Its Applications

**FIGURE 3:**
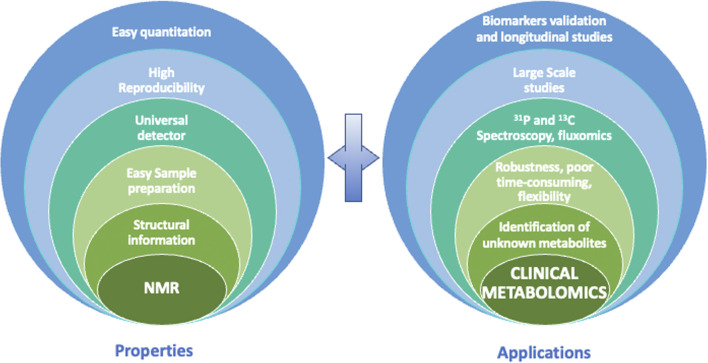
Properties of NMR that allow specific advantages for clinical metabolomics.

Although the limitations of NMR have been briefly stated, we must keep in mind some of its interesting characteristics to understand why NMR is an analytical technique of choice in the field of clinical metabolomics (Figure 3). First, NMR is highly reproducible, and it has intrinsic quantitative properties. Second, NMR is non-selective for analytes whose concentration is above the limit of detection, allowing almost universal detection for all organic molecules, depending of course of the sample preparation applied. Third, it provides crucial structural information owing to the high informative character of chemical shift and J-coupling information contained in NMR spectra. Fourth, NMR is non-destructive, which makes it possible to recover precious samples, and most importantly allows multiple 1D and 2D experiments on a single sample. As shown in Figure 3, all of these properties, which will be detailed above, have a positive impact on clinical metabolomics and enable many valuable NMR-based metabolomics studies.

#### Robustness and Reproducibility

The robustness and high reproducibility of NMR relies on the spectroscopic and physical measurement of samples. This capability is very important in the context of statistical data processing to minimize experimental variability and thus increase the sensitivity of the approach. Furthermore, with an adapted standardization of the acquisition parameters, NMR could also potentially allow the comparison and the integration of datasets from different instruments and/or performed at several sites. This potential will greatly facilitate biomarker validation. Indeed, the comparison of different datasets is one of the challenges that clinical metabolomics have to address in order to improve the quality and the robustness of the results and reach the standard required to enter into clinical practices. Moreover, coupled with automated samples preparator and changers, NMR, and more especially ^1^H NMR, allows high throughput measurements of samples. Thus, this approach is currently the unique analytical platform that is adapted to large scale epidemiology but also to longitudinal studies as described in many recent publications (Jobard et al., 2017; Locci et al., 2018; Sliz et al., 2018; Welsh et al., 2018; Vignoli et al., 2019b; Debik et al., 2019; Deelen et al., 2019). For example, Vignoli et al. analyzed by NMR-based metabolomics the serum samples of 978 patients collected after an acute myocardial infarction and that were clinically followed during 2 years. The aim of this study was to explore if metabolomics profiles of patients could be correlated with a higher death risk and could enhance the existing prognostic risk models of death. Authors demonstrated on both training and validation sets that metabolomics data were relevant to identify high risk patients and that combination of these data with existing scoring methods was able to improve risk classification (Vignoli et al., 2019b). In a same epidemiologic approach, Deelen & al. measure with a standardized high-throughput NMR-based procedure, the metabolome signature of more than 40,000 individuals selected in several European cohorts. The aim to this study was to identify metabolites that could predict long term mortality. Using a stepwise procedure, they identified 14 circulating biomarkers that are independently associated with all-cause mortality. These markers could improve the existing score based on conventional risk factors and could potentially be used to help clinicians to define individual strategy for at risk patients (Deelen et al., 2019). In another recent publication, urine NMR metabolomics has been identified as an interesting pipeline for large-scale epidemiology studies. This study demonstrated that it was possible to quantify 43 metabolites and to assigned more than 100 metabolites using a semi-automated methodology in a 1,004 individuals’ cohort. Intra-assays measurement of metabolites concentrations highlighted that with a coefficient of variation (CV%) less than 5%, urine NMR could provide highly robust and accurate results. However, the authors also reported that, as expected, the intra-individuals’ variations in the metabolites over 30 days, as well as inter-individuals’ variations are very high (respectively CV > 20% and >40% for most of the metabolites). They conclude that high throughput urine NMR-based metabolomics could be an interesting and new base for epidemiologic and genetic applications (Tynkkynen et al., 2019). At the longitudinal level, Jobard et al. were able to follow-up by NMR-based metabolomics two types of treatments of HER-2 positive breast cancer patients (79 individuals) during 13 weeks (6 time-points). With this approach, the authors identified which treatment led to the most relevant impact on the patient’s metabolism and highlighted that this effect is still observable several weeks after the end of the therapy. This work demonstrated that metabolomics could be used to predict clinical response or toxicity and tailored the treatment to patients (Jobard et al., 2017).

#### Detection and Quantitation

NMR is often presented as a universal detector. Indeed, any organic molecule with carbon, phosphorus, nitrogen or protons present in a solution will give a specific NMR signal or signals. Of course, for reasons of sensitivity and chemical and physical characteristics, compounds with one or more protons are the most easily detectable. Thus, within the detection limits of the system, NMR can visualize all the molecules present in a sample, without matrix or ionization effects that could affect the signal of certain compounds, and depending on the sample preparation applied. This property makes it possible to quickly visualize all the samples in a cohort and to easily identify outliers and possible subgroups. This is crucial for a better understanding of the structure of cohorts or groups. Moreover, this “universal” detection can be correlated with one of the most important properties of NMR, namely that it could be intrinsically quantitative. Indeed, not only signal intensity is directly proportional to the concentration of a molecule (taking into account the number of nuclei in the molecule), but if qNMR conditions are ensured, such as full relaxation, sufficient signal-to-noise and proper reference signal, the coefficient of proportionality is the same for all peaks, making it possible to quantify multiple analytes with a single internal or external reference (Holzgrabe et al., 2005). Hence, under controlled spectral conditions, NMR is one of the few techniques that allows quantification without the need for reference compounds or calibration curves. It is probably this feature that makes NMR unique and a tool of choice for clinical metabolomics. It is indeed clear that in the context of personalized medicine, the longitudinal aspect of the metabolomics studies is an essential point especially for patient follow-up and treatment evaluation. Comparison of metabolic profiles over time is not possible without a solid baseline and robust values. Moreover, no biomarkers could be useful without quantitation (Wishart, 2016). For multi-omics integration, as well as for translational purposes and clinical applications, absolute quantification appears as a keystone and a requirement of metabolomics studies (Wishart, 2016; Pinu et al., 2019). Depending on the biofluid or tissue examined, several protocols, recommendations and commercial solutions (such as Bruker IVDr NMR platform) have been proposed that allow quantification of 50–150 metabolites in one experiment through 1D NMR spectroscopy and in a range of concentrations from µM to mM and with a huge reproducibility (Nagana Gowda et al., 2015a; Emwas et al., 2016; Wallmeier et al., 2017; Jiménez et al., 2018; Amiel et al., 2019). This number may appear relatively small compared to the hundreds of metabolites that can be identified in MS, but it should be borne in mind that we are talking about quantification and not just identification. According to the importance of the field, commercial softwares, algorithms and workflows have been recently developed to facilitate automated or high throughput NMR quantification (*i.e.*, Batman, Bayesil, Speaq 2.0, SasMeQ, AQuA, SigMa, ChenomX) (Hao et al., 2012; Ravanbakhsh et al., 2015; Jung et al., 2016; Verhoeven et al., 2017; Beirnaert et al., 2018; Röhnisch et al., 2018; Khakimov et al., 2020). Some recent publications have demonstrated the interest of quantification in clinical metabolomics studies. For example, in a targeted approach, 27 metabolites (mainly amino acids) serum concentrations have been measured in a cohort of 157 smoker’s patients with and without chronic obstructive pulmonary disease (COPD) and have highlighted that reduced amino acid concentrations could be associated with an increase incidence of respiratory exacerbation (Labaki et al., 2019). In a longitudinal study of ischemia reperfusion injury in adult cardiac surgery, NMR-based metabolomics on serum was used to follow-up patients up to 20 h post-operatively. 57 metabolites were quantified to get a longitudinal dataset that allow the exploration of the time-dependent alterations related to surgical trauma (Maltesen et al., 2020). NMR was also used to quantify short chain fatty acid (SCFA) in patients stools and demonstrated good correlations between high levels of SCFA, hypertension and on non-dipping blood pressure profile. This study highlighted the capability of NMR to easily quantify metabolites in stools and to also understand the impact of microbiota on human disease (Huart et al., 2019; Huart et al., 2021a).

#### Metabolite Identification

The development of two-dimensional approaches (COSY, TOCSY, HSQC, *etc*), coupled with methodological advances, should make it possible to increase the number of metabolites that can be detected and quantified (Féraud et al., 2020; Martineau et al., 2020). These developments are further discussed in *Recent and Future Developments in Nuclear Magnetic Resonance-Based Metabolomics*. Interestingly, NMR could also be used to guide MS quantification demonstrating the good complementarity between these two analytical platforms (see *Combining Nuclear Magnetic Resonance to Mass Spectrometry in Clinical Metabolomics*). Moreover, NMR is known to be the technique that provides the most structural information to characterize organic molecules. This can be extremely important for the identification of known or still unknown or undescribed metabolites (Dona et al., 2016; Wang et al., 2019). Accurate identification of metabolites is obviously essential at different levels for the accuracy and the relevance of the all the metabolomics studies. During the last decade, the development of metabolites databases and automated comparison tools increased the possibility to assign metabolites by comparing the NMR data between samples and reference spectra. We can cite HMDB (https://hmdb.ca), which is probably the most complete in terms of metabolites, BNL-NMR database (http://www.bml-nmr.org), BMRB (https://bmrb.io/metabolomics/), Metabolight (https://www.ebi.ac.uk/metabolights/) and some commercial software and platforms that allow the identification and quantification of metabolites (*i.e.*, Bruker IVDr platform and ChenomX software^©^). Many of these databases are interconnected to increase their potency and include more or less complex identification and search automated systems. Table 1 describes the main characteristics of the open access and free databases. Obviously, these spectral databases as well as the comparison, identification and quantification tools still need to be improved by adding new metabolites spectra and data and by the development of more powerful algorithms for the automation of metabolite identification and quantification.

**TABLE 1:** The main free access NMR databases.

Databases	NMR data	Number of metabolites	Automation search	Quant^a^	Others
HMDB	1D and 2D spectra, experimental and raw data, chemical shifts list	>100,000 (not all with measured NMR spectra)	1D and 2D search according to chemical shifts	No	Multiple information about the metabolites (concentrations in different fluids, physical and biological properties, enzymes and transporters, metabolic pathways … ) Very complete
BML-NMR	1D ^1^H and 2D J-resolved spectra, experimental and raw data, different spectral conditions	208	Possible with J-resolved data	Possible with J-resolved data	Many experimental conditions are proposed (experiment type, water suppression, buffer, excitation angle and relaxation delay)
BMRB	1D (proton and carbon) and 2D spectra, experimental and raw data, chemical shifts list	Repository for data	1D and 2D search according to chemical shifts, to mass, to solvent and field strength	No	Many 1D and 2D spectral types, 3D structure. Not limited to metabolites
		Huge number of metabolites			
Metabolight	Mainly 1D proton spectra, spectral experimental conditions and raw data	Repository for data	According to name	No	Database containing many metabolomics datasets
		Huge number of metabolites			

a Quantification.

#### Multi-Nuclei Detection

Even if ^1^H is the most frequently detected atom in NMR-based metabolomics, ^31^P and especially ^13^C can also be used in metabolomics. Even if the sensitivity of ^31^P NMR is less than that of proton, it remains extremely interesting to explore. Indeed, phosphorus is an essential element in the biochemistry of the organisms. Phosphorylation or dephosphorylation of enzymes and proteins via kinases or phosphotransferases plays a key role in many processes, while certain phosphorylated metabolites and enzymes (NAD, NADH, NADP, UTP, CTP, ATP, ADP, AMP, *etc.*) are the mainstays of the energy machinery of cells. Moreover, phosphometabolites would represent more than 30% of the metabolites identified (Mazurek et al., 1997). Phosphorus NMR is still under-exploited at the moment, but because of its specificity, it represents a unique tool that is very interesting to develop for metabolomic applications (Bhinderwala et al., 2020). At the organic level, carbon is undoubtedly the most interesting element to examine, especially in NMR because it is present in all the molecules of interest and its chemical shift range is much wider than that of the proton, which considerably increases its resolution and facilitate the identification of the biomarkers. Unfortunately, ^13^C NMR suffers from a low sensitivity, owing to the low natural abundance and gyromagnetic ratio of ^13^C nuclei. Moreover, quantification is not as straightforward as with ^1^H NMR. Therefore, direct carbon measurement is hardly ever applied in the field of clinical metabolomics. Still, ^13^C spectroscopic information can be obtained with enhanced sensitivity via two-dimensional heteronuclear correlation experiments with inverse detection such as HSQC and HMBC. We have also to mention that ^13^C and sometime ^15^N observations are also very important in fluxomics which aims to quantify fluxes of metabolic reactions and is extremely important in *in vivo* and *in vitro* fundamental studies of biological systems (Crown and Antoniewicz, 2013; Niedenführ et al., 2015; Millard et al., 2017; Giraudeau, 2020). For example, by using labeled substrate (*i.e.*, ^13^C labelled glucose or ^15^N glutamine), this approach allows to follow the evolution of selected biochemical pathways by observing the labeled metabolites that are formed over time. NMR is particularly well suited for monitoring and quantifying the precursors and products of these biochemical pathways. It also allows to easily map the location of stable isotopes and to determine the incorporation points of markers in metabolites (Massou et al., 2007; Lane and Fan, 2017).

#### Sample Preparation

The nature of biological samples analyzed in clinical metabolomics is often complex and sometimes not directly compatible with analytical techniques, especially NMR and MS. This leads to the need to adapt pre-analytical protocols, for example by precipitating proteins, which considerably increases the complexity of the analyses by introducing a significant risk of variability. However, in NMR, it has been possible to develop spectral techniques that limit the pre-analytical steps and thus the manipulation of the samples (*e.g.*, CPMG pulse sequence to suppress protein signals, pre-saturation pulse sequences to suppress the water signal). At this level, NMR is therefore less time consuming and less likely to introduce undesirable experimental variability (Beckonert et al., 2007; Emwas et al., 2016; Vignoli et al., 2019a; Giskeødegård et al., 2019; Snytnikova et al., 2019). The absence of chromatographic techniques gives more flexibility to this approach and allows the rapid analysis of classical biofluids (blood, urine, saliva, cerebrospinal fluid) but also of various samples types (*e.g.*, biopsies, cells, feces, bronchoalveolar lavage fluid) (Ciaramelli et al., 2017; Kim et al., 2018; Romano et al., 2018; Albrecht et al., 2020; Duarte et al., 2020). Due to the nature of the analytical platforms used, most metabolomics experiments require the use of liquid samples. For solid samples such as cells or biopsies, this necessarily involves the insertion of a lysis step in the sample preparation process (Beckonert et al., 2007; Matheus et al., 2014; Kostidis et al., 2017; Mili et al., 2020). This step is sometimes difficult to implement and can lead to a lack of reproducibility and a loss of time. Direct observation of solid or semi-solid samples would limit these drawbacks. The use of high-resolution magic angle spinning (HRMAS) NMR spectroscopy enables the measurement of metabolites in intact tissue or cells and the detection of few dozens of compounds (Gogiashvili et al., 2019; Ruhland et al., 2019; Tilgner et al., 2019). Even if the resolution is lower than in classical high-resolution liquid NMR, this unique application can be particularly interesting at the clinical level, especially as a rapid diagnostic tool (10–15 min) for the analysis of biopsies. Moreover, recent progress in the miniaturization of such approach make it possible to limit its invasive character, hence promising application perspectives in clinics (Lucas-Torres et al., 2021).

### Nuclear Magnetic Resonance-Based Lipidomics

Lipids are a large group of biomolecules, classified, due to their molecular weight, among the metabolites. Different subgroups are often distinguished including fatty acids, glycerolipids, phospholipids, sterols and more specifically, ceramides, sphingolipids, acyl-carnitines, lipoproteins. They play a key role in many biological processes since they can act as energy reservoir, signal molecules, protein traffickers and of course main constituents of plasma membranes (Gross and Han, 2011). Many diseases (such as cancer, diabetes, cardiovascular diseases) and pathological conditions are often accompanied by lipid dysregulation (Lydic and Goo, 2018; Guo et al., 2020). Initially included in metabolomics, the importance of this field, linked to the physicochemical specificities of these compounds, to their very huge number and to their essential biological role quickly led to the appearance of a distinct approach called lipidomics (Dennis, 2009). The analysis of lipids can face several critical issues: 1) the complexity of the samples, the huge number of compounds and their broad diversity of concentrations, 2) their nature and the high number of isomers and isobaric lipids and 3) their physicochemical properties. Mass spectrometry, generally coupled with liquid or gas separative techniques, is currently the analytical technique of choice for lipidome analysis, especially since the development of devices providing additional separation via ion mobility (Paglia et al., 2015; Jurowski et al., 2017; Leaptrot et al., 2019). Due to its lack of sensitivity and resolution, the use of NMR for lipidomics has been limited for a long time to fundamental studies such as 1) determining the structure of lipids of biological interest, 2) studying the structure and the composition of plasma membranes using ^13^C-labeled precursors and ^31^P NMR, 3) monitoring the impact of pathological conditions on lipid metabolism. More recently, NMR demonstrated its ability to be useful in classical lipidomic analyses and to be a very interesting and complementary tool to MS (Li J. et al., 2017; Gil et al., 2019). For example, ^31^P spectroscopy could be chosen to monitor selectively and quantitatively phospholipid classes. Moreover, even if it does not allow, like MS, to finely separate lipids, proton NMR can nevertheless be used to carry out quantitative studies of lipids belonging to the different major classes. Several studies have thus demonstrated the interest of this approach in clinical lipidomics (Ouldamer et al., 2016; Curtarello et al., 2019; Bruzzone et al., 2020; Huart et al., 2021b) and specific workflows and tools (i.e., Lipspin) have been developed for semi-automated profiling (Barrilero et al., 2018; Amiel et al., 2019; Johnson et al., 2021). Recent developments in two-dimensional NMR also open new perspectives to increase the resolution and the identification of lipids (Marchand et al., 2018; Wang et al., 2020). Another specific aspect related to NMR is its ability to profile lipoproteins in blood (Soininen et al., 2015; Kostara et al., 2017; Jiménez et al., 2018). Lipoproteins are supramolecular lipid transporters classified by density ranging from Very Low-Density Lipoproteins (VLDL) to chylomicrons. These particles and more specifically their profile of distribution between the different subclasses is particularly important to measure in different pathological status such as cardiovascular diseases, metabolic syndrome neuropathologies and degenerative diseases (Catapano et al., 2016; Lambert et al., 2020). As described in different papers of the literature, ^1^H NMR is probably the most adapted methodology to obtain fine quantitative profiles of lipoproteins (Jiménez et al., 2018).

### Nuclear Magnetic Resonance Metabolomics and Personalized Medicine

Personalized medicine is a new paradigm in patient care and thus is still under development. It will be based on a better characterization of the patient, his physiological state and his response to treatment. Because it allows the discovery of biomarkers and the stratification of patients, metabolomics, as well as pharmacometabolomics which evaluates their response to treatment, are and will certainly be part, with genomics, transcriptomics and proteomics of the key tools in this new way of approaching pathologies (Wishart, 2016; Jacob et al., 2019; Beger et al., 2020; Ashrafian et al., 2021). Its characteristics of reproducibility, robustness, speed and its ability to quantify metabolites, certainly position NMR as an analytical technique of choice in metabolomics-based personalized medicine, especially because longitudinal aspect is essential (Everett, 2017; Jacob et al., 2019). As mentioned previously, clinical metabolomics and personalized medicine approach are clearly connected. Therefore, most of the recent studies in clinical metabolomics are clearly oriented towards this personalization. Oncology is certainly the field of application where personalized medicine using “omics” is the most advanced (Yu and Snyder, 2016). Indeed, a fine classification of patients, a better evaluation of the efficacy of treatments and a clear vision of prognoses are essential for effective patient management. Thus, several recent studies and papers have demonstrated that NMR-based metabolomics can be effectively applied to precision oncology (Palmnas and Vogel, 2013; Hu et al., 2021; Vignoli et al., 2021).

### Fingerprinting Approach and Application in Clinical Biology

Among all the possible approaches developed in metabolomics, fingerprinting is probably the one that has been applied first. It refers to the non-targeted and without a priori metabolomics studies that led to the identification of specific spectral or chemical patterns that could be related to a pathological or a particular status without identification of the metabolites (Kosmides et al., 2013). This approach is obviously not incompatible with the identification and quantification of biomarkers, but it is focused on a more holistic view of the metabolome and its possible transformation over time or under the effect of a pathology. The possible diagnostic application of fingerprinting is immediately obvious. However, it is equally obvious that such an application inevitably implies reproducibility, robustness, standardization of analytical methods and capability for high throughput analyses as it is the case in clinical biology. These are precisely among the strengths of NMR. As previously mentioned, NMR is indeed particularly well adapted to the study of large cohorts and thus to fingerprinting (Amathieu et al., 2014; Rzeznik et al., 2017; Takis et al., 2019). This approach faces several challenges and requires obviously the development of specific workflows and methodologies, especially for the multivariate analyses of the raw data (Zacharias et al., 2018; Markley et al., 2019). Besides the diagnostic models that metabolomic fingerprints can generate, it could also be very useful in a preventive framework, essential in the personalized approach to treatment. Indeed, regular observation of the metabolomic profile of patients would undoubtedly allow early identification of deviations that could be linked to the onset of certain pathologies (Takis et al., 2019).

Another important question concerning NMR-based metabolomics is its interest and its capacity to become one of the tools used in clinical practice and its interest compared to the techniques used until now in clinical biology. The numerous examples found in the literature demonstrate the strong potential of metabolomics in clinics, but the transition from laboratory to clinical practice is still a major challenge (Pinu et al., 2019). It can only be filled by the transition of metabolomics to the standards of quality, robustness and reproducibility required in clinical biology and NMR certainly has an important role to play (Ashrafian et al., 2021). It is clear that one of the first clinical applications of metabolomics remains the discovery of new biomarkers but that this approach, because of its holistic aspect, can bring much more to the understanding of pathologies, the prediction of their evolution, the stratification of patients and the evaluation and adaptation of treatments. Far from competing with existing tools, clinical metabolomics, once it masters and standardizes its analysis and data processing protocols, will undoubtedly provide essential information for improving patient care. NMR, thanks to its analytical qualities, its robustness and its ease of automation is undoubtedly a technological platform that will find its place among other instruments capable of providing clinicians with the data necessary for diagnosis and monitoring of patients.

For the sake of completeness, we should also highlight *in vivo* Magnetic Resonance Spectroscopy (MRS) which consists in localized NMR spectroscopic acquisitions performed within Magnetic Resonance Imaging (MRI) systems. It is the only technique that allows *in vivo* investigation of the human metabolome. (Rhodes, 2017; van de Weijer and Schrauwen-Hinderling, 2019). In this review, we have focused on classical high-resolution NMR spectroscopy without detailing *in vivo* or imaging applications, which are vast areas of clinical interest and would deserve a dedicated review. We will describe in the following sections how NMR limitations have been or will be challenged, what are the methodological and technological evolutions that will allow NMR to evolve in the near future and to remain a powerful analytical platform in metabolomics. We will also examine how this technique can be advantageously combined with other analytical approaches and why this complementarity may represent a solution for improving our knowledge and exploration of the metabolome.

## Combining Nuclear Magnetic Resonance to Mass Spectrometry in Clinical Metabolomics

The previous section demonstrated the strong potential that NMR spectroscopy has within the field of clinical metabolomics. However, it is well-known that NMR-based metabolomics has some drawbacks, namely its lack of sensitivity and the non-negligible signal overlap in routine 1D ^1^H experiments of complex biological samples. This limits its application within several fields, including personalized medicine. Indeed, signal overlap makes the difficult task of metabolite identification and the subsequent biomarker discovery even more difficult. As such and as explained previously, mass spectrometry based metabolomics became more popular than NMR spectroscopy in a vast majority of metabolomics applications (Letertre et al., 2021). However, MS techniques come with their set of drawbacks as well: lack of robustness and repeatability, and the difficulty to identify the biomarkers corresponding to the numerous features detected in MS spectra. These drawbacks are not to be ignored in clinical research, as the discovery of biomarkers of a given pathology, or biomarkers of an exposition to a therapeutic treatment, request robust and repeatable methods for intra and inter-laboratory comparison. To overcome the respective drawbacks of each techniques and to combine their strengths, the complementarity between both NMR spectroscopy and MS-based metabolomics techniques has been discussed several times in the past 15 years (Pan and Raftery, 2007; Marshall and Powers, 2017; Letertre et al., 2021). In the following section, different examples of studies combining both NMR and MS-based metabolomics applied in clinical settings are presented, and their advantages and drawbacks are discussed.

### Nuclear Magnetic Resonance Hardware Hyphenation to Mass Spectrometry Hardware

Combining NMR with liquid chromatography (LC-NMR) and further with MS (LC-NMR-MS) through hardware hyphenation has been long done, especially in natural products analysis and the different ways of doing it have been nicely described recently (Gebretsadik et al., 2019). This approach has also been found useful for drug metabolism (Shockcor, 2002) and pharmaceutical research (Lindon et al., 2000; Lindon et al., 2002). For instance, the combination HPLC-NMR with an ion-trap MS made the identification of paracetamol metabolites and endogenous compounds in human urine possible (Shockcor et al., 1996). By successfully detecting phenylacetylglutamine, which was not possible by using only ^1^H NMR, this triple-hyphenated system overcame the NMR signal overlap issue. One the other hand, the NMR part of the system was an essential tool to determine which isomers of the paracetamol-glucuronide conjugate was present in the sample (Shockcor et al., 1996). A similar investigation applied this system to characterize ibuprofen metabolism in human urine (Clayton et al., 1998). LC-NMR-MS was also used in parallel to ^19^F NMR spectroscopy to investigate the metabolism of fluorinated novel drug candidates (Dear et al., 1998) or drug intermediates (Scarfe et al., 1999; Scarfe et al., 1998) within urine samples of animal models and without requesting specific radiolabeling. However, the community has lost interest in LC-NMR-MS since the past decade, most certainly due to the technical difficulties encountered by combining techniques coming with orthogonal analytical requirements (Silva Elipe, 2003).

### Combining Nuclear Magnetic Resonance and Mass Spectrometry Datasets

#### To Increase Metabolic Coverage

Rather than hyphenating their respective hardware, combining the datasets of NMR and MS-based metabolomics workflows has had more success. The most obvious reason to use both NMR and MS-based metabolomics is to increase the metabolic coverage, thus increasing the chance of identifying new biomarkers. Indeed, it is well emphasized within the metabolomics community that no tools whatsoever offer a full coverage of the metabolic landscape. Several studies nicely supported this assessment by using a Venn diagram, which shows how the metabolite identification overlays between the different platforms used. One of the most famous example is a study of Human Serum Metabolome (Psychogios et al., 2011). By using five analytical platforms (NMR spectroscopy, LC-ESI-MS/MS, GC-MS, DFI-MS and TLC-GC-FID), the authors were able to identify 3,764 compounds, from which only 200 were commonly detected by at least two platforms. Furthermore, this effort was completed by quantitative data for some of the detected metabolites, and although some of the results differed between platforms, good agreement overall were found (Psychogios et al., 2011). The combination of GC-MS, LC-MS and NMR was also applied to explore a NIST standard reference material for human plasma and its application in clinical laboratories. A total of 353 metabolites were identified, and whilst GC-MS was the analytical technique showing the most of unique identification (65), and that LC-MS and NMR identifications were found to overlap, NMR still allowed to detect small sugars which were not directly accessible by LC-MS (Simón-Manso et al., 2013). Similar to the Human Serum Metabolome, a study focused on the mouse skeletal muscle metabolome by combining NMR, FIA-MS, GC-MS and LC-HRMS, highlighted 132 discriminant metabolites, from which only 17 were detected by more than one analytical platform (Bruno et al., 2018). Importantly, the analytical approach proposed in this article was aimed to be easily adapted for human clinical trials. In a final example, the effect of therapeutic treatment on human gastric cancer cells was assessed by metabolomics and lipidomics by using three analytical platforms (NMR spectroscopy, GC-MS and LC-MS). Once again, out of the 111 compounds detected, only 21 were commonly highlighted by at least two analytical techniques (*Goulitquer et al., 2018*). This proves the importance of using multiple platforms if the aim of a given study is to capture the metabolome as broadly as possible, or to carefully chose the appropriate platform if only a specific subpart of the metabolome is of interest, as the different requirements in term of sample preparation as well as the very essence of the analytical platform selected will give access only to a limited part of the metabolome.

#### To Correlate Variables Detected by Nuclear Magnetic Resonance and Mass Spectrometry Techniques

**FIGURE 4:**
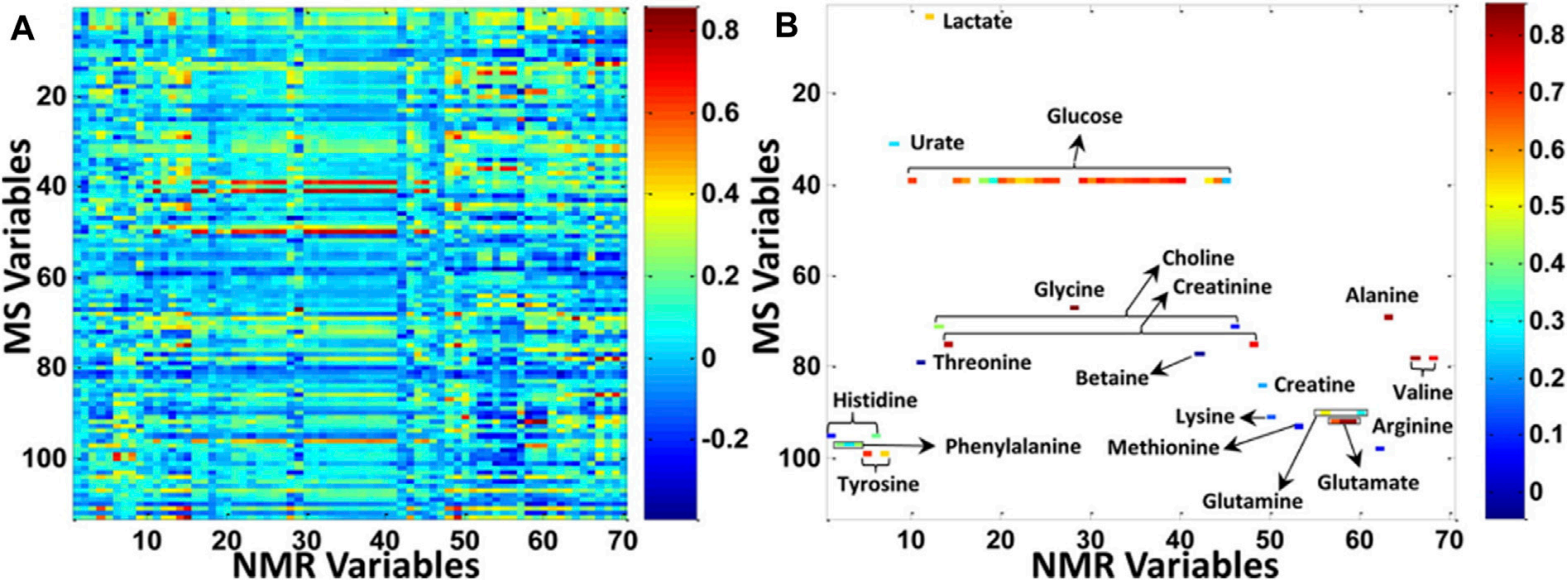
Increasing the metabolic coverage of serum samples from patients suffering from colorectal cancer and polyps, by using both NMR and MS analytical platforms. **(A)** Correlation between all NMR and MS variables. **(B)** Correlation between the subset of metabolites (labeled in the figure) that can be detected by both NMR and MS. The *X* axis provides an index of all NMR variables in the data matrix, and the *Y* axis provides an index of all MS variables in the data matrix. Reproduced with permission from (Deng et al., 2016).

Another way to show the limited overlap that can be observed between NMR and MS datasets is to correlate their respective signals, as it was done in a study focusing on colorectal cancer and polyps serum samples that were analyzed by NMR spectroscopy and targeted LC-MS/MS (Deng et al., 2016). It is clear that in Figure 4B, the correlation taking into consideration only the variables that were commonly detected by both NMR and MS techniques represented only a small subset of all the features detected either by NMR or MS (Figure 4A). However, correlating the intrinsic covariance of signals detected by each of the analytical techniques can also serve as a tool to confirm metabolite annotation made by one analytical technique, or to acquire deeper knowledge on biomolecular reactions and thus to enhance biomarker discovery. The first tool based on this methodology was the so-called SHY (statistical heterospectroscopy), based on a Pearson correlation method. Crockford *et al.* showed positive or negative correlations between NMR with LC-MS signals measured within urine samples of rats treated with hydrazine as a proof-of-concept (Crockford et al., 2006). This approach was further applied to human urine samples, for instance to highlight biological processes of inborn errors of metabolism by correlating NMR and DESI-MS signals (Pan et al., 2007) or to successfully investigate the xenometabolome of a random subset of an epidemiological study (Crockford et al., 2008). In this last study, new drug metabolites were discovered thanks to the correlation between NMR and MS signals but also to the use of MS tools to investigate ion fragmentations, such as MS^E^ and different collision energies (Crockford et al., 2008).

#### To Improve Statistical Models Through Multi-Block Data Integration

The second advantage of combining NMR and MS-based metabolomics datasets is to produce more robust and trustworthy multivariate statistical models. To do so, multi-block data fusion, or data integration, is gaining in popularity within the metabolomics community (Doeswijk et al., 2011; Boccard and Rudaz, 2014). Three levels are available to apply data integration, often referred as low-, mid- and high-levels. The difference between these levels have been clearly explained previously (Boccard and Rudaz, 2014). Briefly, low-level data fusion consists in taking the matrices obtained by each of the analytical technique as they are, without performing multivariate statistics on the individual blocks beforehand. Mid-level consists in reducing the individual matrices before integration (*e.g.*, by selecting the most discriminant variables), and high-level data fusion integrates only the global outputs of the individual statistical models. Once the fusion matrix has been produced, chemometrics can be applied, in a very similar way as for the individual matrix, by using unsupervised and supervised statistical analysis (Boccard and Rudaz, 2014).

In a first example which aimed at determining metabolic differences between serum samples from breast cancer patients and healthy controls, a mid-level data fusion approach was used to enhance the discriminative performance of unsupervised analyses and limit the misclassification of the supervised analyses performed on the individuals NMR and the direct analysis in real time (DART-MS) models (Gu et al., 2011). In that end, another supervised analysis was performed by setting up the Y variable to the first component of the unsupervised NMR model, which performed better than the MS model, and the X matric was set as the DART-MS dataset, containing more variables. The resulting model performed better in term of both discriminative and misclassification performances. However this is not always the case, as shown in another example where the discriminative power of supervised models based on the combined NMR and GC-MS datasets did not outperform the supervised models of the individual datasets (Teul et al., 2009). Still, correlations between the discriminant features of the multi-block model, which were detected by both the NMR and GC-MS datasets, offered a better understanding of the metabolic alterations lying in the plasma samples of patients suffering from stable carotid atherosclerosis compared to controls (Teul et al., 2009). This proves the benefits of combining those two techniques to either help increasing the metabolic coverage, improving multivariate analysis performance or have a deepest understanding of the biological processes.

This is also well exemplified in a study which investigated the metabolic profiles of human dopaminergic neuroblastoma cells treated with different neurotoxins and analyzed by both NMR and high-throughput direct-ionization electrospray ionization MS (DI-ESI-MS) (Marshall et al., 2015). Firstly, the sample preparation was optimized to propose a dual analysis of the same sample which thus prevented extra sample handling. Secondly, the discriminant power of the multi-block principal component analysis (PCA) model which integrated the NMR dataset with the DI-ESI-MS dataset through a low-level approach, was clearly higher than the single NMR or DI-ESI-MS-based PCA models. Finally, the metabolite identification of the discriminant features were facilitated by accurate mass measurements and fragmentation patterns obtained by tandem MS experiments (Marshall et al., 2015). From top to bottom, this demonstrates how it is possible to successfully combined NMR and MS dataset to explore the effect of a specific treatment relevant to Parkinson’s disease, and further clinical applications will most certainly be observed in the coming decade. However, careful attention should be paid to single block formatting during multi-block integration, especially through a low-level approach. Indeed, the aim is not to give too much weight to some variables or to one of the block considered for the integration (Boccard and Rudaz, 2014). In the study discussed previously combining NMR with DI-ESI-MS, each block were scaled to unit variance and by the square root of its variable count (Marshall et al., 2015).

**FIGURE 5:**
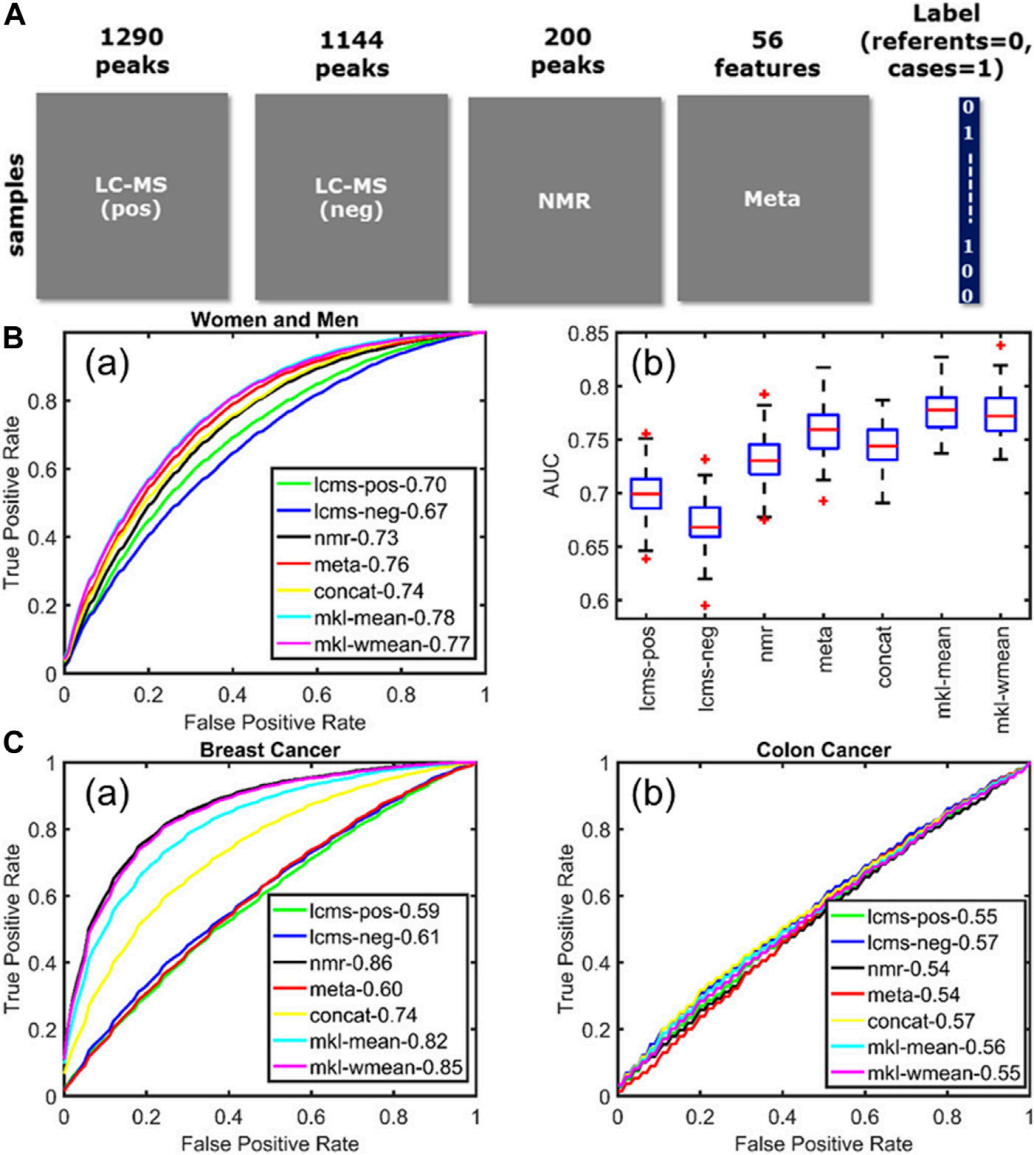
Multiblock data fusion applied to study the metabolic alterations in human plasma samples from patient suffering from chronic diseases. **(A)** Data sets used in this study: metabolomics measurements (LC–MS and NMR), the metadata set containing life-style information, and the label information corresponding to each sample. **(B)** Acute coronary syndrome. **(a)** Average ROC curves showing the forecasting performance of individual data sets as well as fusion methods for women and men. **(b)** Boxplots summarize the performance of different approaches across 100 training/test sets. **(C)** Average ROC curves illustrating the forecasting performance of **(a)** breast cancer and **(b)** colon cancer. Figure reproduced with permission from (Acar et al., 2017).

As highlighted by the above examples, data integration has a lot of potential in clinical metabolomics, as the NMR and MS datasets can be fused with metadata describing life-style factors from large human cohorts. This was done in a study which aimed at observing metabolic alterations in human plasma samples from patient suffering from three different chronic diseases (acute coronary syndrome, breast and colon cancers) (Acar et al., 2017). The samples were analyzed by NMR and LC-MS (positive and negative ionization) and the integration of these three blocks with the metadata (Figure 5A), by using multiple kernel learning, provided a model which outperformed the individual models when it came to acute coronary syndrome (Figure 5B). However, the fusion of the different datasets did not improve the performance of the individual NMR model for the breast cancer samples, and of none of the individual models for the colon cancer samples (Figure 5C). In fact, the integration of the metadata can be useful, as it can help picking up novel confounding factors, as it was shown for metabolites linked to coffee consumption and smoking habits (Acar et al., 2017), but these very same confounding factors can also influence the selection of discriminative variables. This is a problem often encountered in metabolomics and even though several methods have been proposed to optimize the variable selection step, such as the one based on sparse multi-block PLSR for biomarker discovery (Karaman et al., 2015) or backward variable elimination from PLS-DA models combined with Monte Carlo Cross-Validation (Deng et al., 2016), this issue remains a current limitation of data fusion.

### Using Nuclear Magnetic Resonance and Mass Spectrometry Strengths to Help Metabolite Identification

Increasing metabolic coverage and sensitivity also means more biomarkers to identify, which is obviously of major interest to understand their roles in specific diseases. The complementarity of the information that can be gathered by both NMR and MS-based techniques represents also an advantage when it comes to identifying biomarkers, especially when high resolution MS (HRMS) is used to acquire accurate mass measurements from parent compounds and their fragments in addition to the structural information obtained by 1D and 2D NMR spectroscopy. Detailed approaches to combine both have actually been proposed, such as SUMMIT MS/NMR (Bingol et al., 2015) or NMR/MS translator (Bingol and Brüschweiler, 2015a). The first one relies on HRMS measurements of a complex sample, from which putative molecular formulas and scaffolds can be proposed and NMR spectra predicted. Those predicted spectra are then compared to experimental HSQC NMR spectra, which have been deconvoluted for each of the sample metabolites (Bingol and Brüschweiler, 2017). To put it simply, NMR/MS Translator could be seen as the reverse of SUMMIT MS/NMR, as it starts with 1D and 2D NMR acquisition, so putative annotations can be made by comparing the experimental NMR spectra to databases. For the best hits, the MS spectra are predicted and then compared to experimental MS spectra (Bingol and Brüschweiler, 2015a). This last approach allowed the authors to identify new human urine metabolites which had never been reported previously. It has also been suggested by the same group that the SUMMIT MS/NMR approach could be applied on analytes which remained unidentified even following the application of the NMR/MS translator approach (Bingol and Brüschweiler, 2017). Upon the fact that some of the steps of these two approaches need to be automated in order to make the entire process more rapid, it could promote the identification of biomarkers in clinical research (Bingol and Brüschweiler, 2015b), but this has not really been widely applied yet.

### Combining Nuclear Magnetic Resonance and Mass Spectrometry Techniques in a Quantitative Approach

Once the strengths of NMR and MS have been joined to increase the metabolic coverage, to provide more powerful statistical models and to identify new metabolites, new biomarkers of interest can be highlighted. However, approaches combining NMR and MS datasets often considers relative concentrations. Needless to say, that this is not satisfactory for clinical applications and on the contrary, absolute concentrations are needed for intra- and inter-laboratory comparison, as well as to compare data obtained with different analytical strategies. Recent methods have been proposed toward that goal. One, called « NMR-guided-MS quantitation », which consist in acquiring the absolute concentrations of analytes present in a randomly selected reference sample by NMR, which are then used as concentrations of reference for the rest of the samples analyzed by LC-MS/MS (Nagana Gowda et al., 2018). This method was applied to quantify 30 human serum metabolites in eight samples, and showed excellent correlations between the concentrations obtained by NMR and the ones obtained by NMR-guided MS, and good agreement between the NMR-guided MS approach and stable-isotope-labelled internal standards (SIL IS) measurements by MS. However once considering each of the metabolites individually, even though most of them showed good correlations between NMR and NMR-guided MS (*e.g.*, *R* ^2^ = 0.989 for proline), some demonstrated very poor correlations (*e.g.*, *R* ^2^ = 0.207 for pyroglutamic acid) (Nagana Gowda et al., 2018). This needs to be seriously consider when it comes to clinical biomarker discovery as whatever explanation lying behind those poor correlations (*e.g.*, glutamine cyclization (Purwaha et al., 2014; Nagana Gowda et al., 2015a; Nagana Gowda et al., 2015b), multiple or poor signals, ion suppression … ), it proves that it is wrong to assume that MS can provide stable measurements of all metabolites. To identify those unstable metabolites, or as an alternative to labour-intensive calibration curves, the NMR-guided MS can be of interest. Build on this approach, another one has been proposed, based on the derivatization of the reference sample with SIL IS and of the rest of the samples with unlabelled IS (Fei et al., 2019). This new approach, called the qNMR-MS, offers the possibility to reduce matrix effect but presents the drawback of adding additional sample handling, potentially limiting when large number of samples are considered.

### Nuclear Magnetic Resonance and Mass Spectrometry Techniques as the Keystones of Fluxomics

A branch of metabolomics which combines both analytical platforms which gathered a lot of interest in clinical research is Stable Isotope-Resolved Metabolomics (SIRM), also referred as fluxomics analysis. SIRM offers the possibility to quantitatively apprehend metabolic pathways and fluxes by measuring isotopomers, by NMR, and isotopologues, by MS, following labelling of a precursor molecule with stable isotope tracers. Most importantly, one of the advantages of fluxomics is that it can be done either *in vitro* or *in situ*. SIRM has thus the potential to lift the veil on the metabolic mechanism of numerous diseases, especially cancer (Lane et al., 2011; Lane et al., 2016; Lane et al., 2019). More active glycolysis and Krebs cycle, as well as an activated pyruvate carboxylation were for instance found to promote tumour development in lung tissues (Fan et al., 2009). The deep gain in knowledge on how diseases actually work can clearly improve personalized treatment (Fan et al., 2012). SIRM is probably the metabolomics branch presenting the finest achievements in combining NMR and MS analytical technologies to unravel disease understanding, but also the most complex one. However tremendous efforts have been done to promote rapid development of new computational tools to help SIRM and other metabolomics branches to be implemented in the long term within clinical laboratories. These computational tools will also certainly help the integration of SIRM findings, or metabolomics in general, with other kinds of datasets, such as genomics, transcriptomics, proteomics or clinical metadata to acquire a more in-depth knowledge of biomolecular mechanism of a pathology.

### Combining Nuclear Magnetic Resonance and Mass Spectrometry Techniques for Personalized Medicine: Where Do We Stand?

Some studies apply both NMR and MS-based analytical strategies to clinical research to combine the respective biomarkers of interest, and include them in a common metabolic pathway analysis, as it was done to study the primary membranous glomerylonephritis and the subsequent nephrotic syndrome that it can cause in adults (Taherkhani et al., 2019). Others use NMR as a primary tool for open-profiling metabolomics, and then use subsequent LC-MS/MS to confirm the results obtained by NMR or to quantitatively target a subset of metabolites. This approach was used to analyze 244 human serum samples from the ECLISPE study and to identify biomarkers of chronic obstructive pulmonary disease (Ubhi et al., 2012), or to analyze 32 neonate urine samples and identify biomarkers related to late-onset sepsis (Sarafidis et al., 2017). Through an example of large epidemiological study performed on 4,680 urinary samples from the INTERMAP study, ^1^H NMR was also used for metabolic phenotyping before applying GC-MS and LC-MS/MS to analyze the urinary amino acids (Chan et al., 2017). Surprisingly when it comes to large epidemiological cohorts, in the 47 studies reported in the COMETS initiative (Yu et al., 2019), relatively few are applying both NMR and MS-based metabolic profiling approaches. From those, it is worse mentioning the AIRWAVE study (Elliott et al., 2014), the MAC study (Chow et al., 2017), the MESA study (Bild et al., 2002) or the TwinsUK study (Moayyeri et al., 2013). The same observation can been done when it comes to metabolomics biomarkers from acute respiratory distress syndrome, chronic obstructive pulmonary disease and asthma (Bowler et al., 2017). In another review focusing on preeclampsia, 16 metabolomics studies were based on MS-data and 12 by NMR, but none were employing both (Kelly et al., 2017). However and as nicely pointed out in this review, combining both could provide more robust and accurate preeclampsia metabolic profile, as the metabolic coverage accessible with each methods in the studies reviewed were not always comparable, also because of the targeted approach often applied in MS which focus only on a subset of the metabolome (Kelly et al., 2017). The integration of NMR and MS metabolomics with other OMICS analytical platforms gathered a lot of interest in the last 3 years in the field of biomedical sciences (Manzoni et al., 2018), personalized medicine (Jacob et al., 2019), environmental health (Yao et al., 2019), microbiome research (Zimmermann et al., 2019) or toxicology (González-Ruiz et al., 2019). Integration of different OMICS platforms have even been of interest for personalized medicine in human space flight (Schmidt and Goodwin, 2013). Furthermore, optimized extraction protocol to analyzed on the same sample the metabolites, the proteins and the lipids have been proposed (Coman et al., 2016), so upon further computational development, the perspectives of integrating NMR-based metabolomics with MS-based metabolomics or other OMICS will certainly promote its application within clinical settings.

## Recent and Future Developments in Nuclear Magnetic Resonance-Based Metabolomics

The first section of this review highlighted the major role that NMR plays as an analytical tool in clinical metabolomics. The second part described how this role can be further strengthened by combining NMR with other analytical techniques, especially MS-based metabolomics. Nevertheless, there are still major challenges posed to the NMR spectroscopists in order to further improve the potential of NMR spectroscopy within clinical metabolomics Indeed, NMR has well-known limitations. As mentioned in the previous section, the main limitation is a reduced sensitivity compared to other analytical methods and particularly MS. The sensitivity of NMR at high magnetic field (>500 MHz) is in the micromolar range. This is sufficient to detect major metabolites in biofluids or extracts, but relatively large sample amounts are often required, and the detection of less concentrated, specialized metabolites can be a challenge. A second limitation arises from resolution issues, since it can be difficult to separate overlapping metabolite signals in crowded spectral regions of the ^1^H spectrum. Finally, a third reason why NMR is less used than MS in the clinical world is the relatively high purchase cost of NMR instruments (>1M€ for a 600 MHz spectrometer) and the associated consumption of cryofluids.

NMR spectroscopy would certainly be much more widely used in the clinical world if the above-mentioned limitations were circumvented. While these challenges are not new, several recent (<10 years) methodological advances in the NMR community have laid the foundations for a more sensitive, better resolved and more accessible NMR spectroscopy (Giraudeau, 2020). This part focuses on such recent advances, which, in addition to the high robustness of NMR spectroscopy, have the potential to provoke a significant paradigm shift regarding the role of NMR for biomedical applications. Some of them have already proved their usefulness in the field while other rather offer mid-term perspectives, but in our view, all are of interest to the fields of clinical studies and personalized medicine.

### Improving the Sensitivity

The sensitivity of NMR directly results from the level of nuclear polarization, generally determined by a Boltzmann law at thermal equilibrium. This results in relatively weak nuclear polarization levels, for instance, only 0.000008 for ^1^H at 300 K in a 14 T magnetic field -the typical NMR metabolomics configuration. The most direct -but technologically challenging-approach consists in increasing the static B_0_ field, since the sensitivity increases with B_0_^3/2^. NMR metabolomics experiments are typically performed between 500 and 800 MHz, but magnets up to 1.2 GHz are now commercially available, providing impressive results on biofluids (Banci et al., 2019). However, such very high field magnets only provide a modest sensitivity gain (a factor 2.8 between 600 MHz and 1.2 GHz) while their cost is at least ten times higher. On the hardware side, more promising perspectives probably arise from the development of more sensitive NMR probes. Cryogenically cooled probes can provide a signal to noise ratio (SNR) improvement by a factor 3 to 4, however they are expensive and show limited efficiency for samples with high salinity (Kovacs, 2005). On the one hand, higher field and cryoprobes are well suited to improve the limit of detection for a given sample volume, but on the other hand, numerous small volume probes have been developed to analyze mass-limited samples without compromising on sensitivity. These include microprobes that can accommodate sample volumes of a few tens of µL (Clendinen et al., 2014), but also recent microfluidic-based probes that can detect metabolites at sub-millimolar concentrations in sample volumes of *ca.* 2 µL (Finch et al., 2016). The incorporation of such microfluidic devices in NMR experiments also makes it possible to perform flow experiments, opening great avenues for time-resolved metabolomics. Patra *et al.* recently applied this approach to non-invasive metabolomic monitoring of microfluidic cultures with as few as 1,250 individual cells (Patra et al., 2021).

In addition to such magnet and probe advances that will certainly enhance the performance of clinical NMR metabolomics, great promises arise from hyperpolarization methods, which have been the focus of many exciting developments in the NMR community in the last 2 decades. Indeed, these approaches can enhance the sensitivity of NMR spectroscopy by up to four orders of magnitude by enhancing the nuclear polarization to values close to unity. The two most popular methods for hyperpolarization are para-hydrogen induced polarization (Duckett and Mewis, 2013) and dynamic nuclear polarization (Plainchont et al., 2018). Both have been discovered many decades ago, but only recent developments have made them applicable to the analysis of complex samples with metabolomics relevance.

The first approach, para-hydrogen induced polarization, is based on the transfer of hyperpolarization from H_2_ in the para state to the nuclear spins of analytes (Duckett and Mewis, 2013). While the initial approach involved a chemical hydrogenation reaction, it was made more versatile and general by the development of the SABRE technique (signal amplification by reversible exchange) which involves the addition of a metal-based complex to reversibly transfer the hyperpolarization to the analytes (Lloyd et al., 2012). This method is very attractive for practical applications since it is simple and relatively cheap. However, it has a certain degree of selectivity since the SABRE catalyst mainly binds to compounds containing electron-donating heteroatoms such as nitrogen. SABRE-based hyperpolarization has already been successfully applied to quantify metabolites in natural extracts (Hermkens et al., 2016). Although it has not yet been applied to a metabolomics study, Tessari and co-workers were recently able to detect numerous metabolites at nanomolar concentrations in solid phase extracts of urine, which forms a promising perspective for metabolomics (Sellies et al., 2019).

**FIGURE 6:**
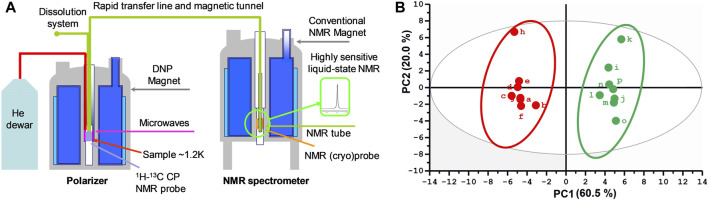
Potential of dissolution dynamic nuclear polarization (d-DNP) for highly sensitive ^13^C NMR metabolomics at natural abundance. **(A)** d-DNP experimental scheme, where the sample is first hyperpolarized at liquid He temperature in a glass-forming solvent by microwave irradiation of free radicals, then rapidly transferred through a magnetic tunnel to a classical NMR spectrometer where conventional detection occurs. **(B)** First application of this experimental scheme in a natural abundance ^13^C metabolomics study, reported in 2020 by Dey *et al.* Scores plot of the principal component analysis (PCA) obtained from 40 spectral buckets from hyperpolarized ^13^C spectra of 16 tomato fruit extracts at different ripening stages (green vs red). Integrals were normalized to Na-TSP-d_4_ as an internal reference and to the weight of the sample used for extraction. Mean centering and unit variance scaling were used in PCA. **(B)** Reproduced from (Dey et al., 2020).

Parallel developments in the NMR world have been focusing on another hyperpolarization technique, dissolution dynamic nuclear polarization (d-DNP, Figure 6A) proposed in 2003 by Ardenkjaer-Larsen and co-workers (Ardenkjaer-Larsen et al., 2003). This approach consists in mixing the sample with small amounts of free radicals, freezing it in a glass-forming solution at liquid Helium temperature and in a static magnetic field, then irradiating it by microwaves at the Larmor frequency of the unpaired electrons. Under such conditions, the very high polarization of the electrons is transferred to nuclei, leading to polarizations close to unity within a few minutes. The frozen sample is then rapidly transferred to a nearby NMR spectrometer, where classical spectra can be obtained with sensitivity enhancements by up to four orders of magnitude. The d-DNP approach is technically demanding but very general, since all metabolite signals can be enhanced in a non-selective fashion. A more fundamental limitation arises from the decrease of hyperpolarization during sample transfer, which occurs as a function of nuclear longitudinal relaxation times (T_1_). As a consequence, most applications of d-DNP have been focusing on ^13^C nuclei, since their T_1_ can reach several tens of seconds, especially for quaternary carbons. In the MRI community, d-DNP has rapidly had a great impact on metabolic imaging, with the first injection of hyperpolarized pyruvate to humans in 2013 (Nelson et al., 2013), and not less than 25 undergoing clinical trials reported in 2019 (Ardenkjaer-Larsen, 2019). In NMR spectroscopy, d-DNP has also been widely used to investigate metabolic processes in real-time, for instance to get insight into enzymatic kinetics (Wilson et al., 2010). In this context, the application of d-DNP to extracts or biofluids opens promising perspectives to enhance the sensitivity of NMR metabolomics, and first steps towards this goal have been reported recently. In 2015, Dumez *et al.* showed that d-DNP could be applied to enhance the ^13^C NMR signals in plant and cancer cell extracts at natural abundance (Dumez et al., 2015), and in 2016, the very good analytical repeatability (<4%) of the method was demonstrated (Bornet et al., 2016). Lerche *et al.* also reported a complementary approach relying on the incubation of the targeted biological material with a^13^C-labeled substrate (Lerche et al., 2017). More recently, in 2020, Dey *et al.* demonstrated, on the example of plant extracts, the first hyperpolarized metabolomics study at natural ^13^C abundance (Figure 6B) (Dey et al., 2020). While these recent methods have not yet been applied to clinical metabolomics, they could pave the way towards the detection of biomarkers that were not accessible by NMR so far. In particular, ongoing technological developments to accelerate the sample transfer (Bowen and Hilty, 2010) -thus making d-DNP compatible with ^1^H detection- and to increase the lifetime of hyperpolarized samples (Ji et al., 2017) could help spreading this promising approach in the metabolomics community.

### Improving the Resolution

Typical samples of metabolomics relevance are extremely complex, since they contain a great diversity of metabolites. Although NMR is not as sensitive as MS, resulting spectra can be extremely complex and characterized by strong and numerous peak overlaps that can alter the high analytical performance of NMR. In a classical untargeted metabolomics workflow, several NMR signals pertaining to different metabolites can be observed within a single bucket, making it difficult to identify relevant biomarkers. When quantitative data are being sought, the accurate determination of peak areas is hampered by such overlaps, leading to errors in concentration determination. Signal processing methods can help deconvoluting individual metabolite contributions, but such methods often rely on databases which are specific of a given matrix prepared under specific conditions (Hao et al., 2012; Lacy et al., 2014; Ravanbakhsh et al., 2015). One can also rely on the detection of heteronuclei, such as ^13^C, that offer a much broader chemical shift dispersion compared to ^1^H (Clendinen et al., 2014). However, due to a limited sensitivity, the routine application of ^13^C NMR metabolomics will require highly sensitive detection methods such as those reported in the previous paragraphs. Fortunately, in addition to these approaches, many innovative NMR methods -based on pulse sequence developments-have been developed to simplify the analysis of complex mixtures, that were successfully transferred to metabolomics in recent studies, and there is not much doubt that at least some of them will become part of the daily clinical metabolomics workflow in a near future.

The most widely used strategy to better separate overlapping signals in NMR of complex mixtures is to rely on multi-dimensional methods such as 2D NMR. Indeed, in 2D NMR spectra, peaks are spread along two orthogonal dimensions (typically ^1^H–^1^H or ^1^H-^13^C), hence reducing peak overlap while providing crucial information on atomic connectivity. 2D NMR has been used for decades to elucidate the molecular structure of chemical compounds, including metabolites. In metabolomics studies, popular experiments such as J-resolved spectroscopy, COSY (correlation spectroscopy), TOCSY (total correlation spectroscopy) or HSQC (heteronuclear single-quantum correlation) are generally applied to a subset of samples from a given study, or to a purified fraction of a biological matrix, to elucidate the structure of biomarkers (Mahrous and Farag, 2015). However, the systematic use of 2D NMR peaks volumes as a raw data in metabolomics workflow is still far from routine. Nevertheless, this would provide a great way to better extract individual metabolite variations, since 2D peaks are much less prone to overlap than their 1D counterparts. Indeed, early studies have shown the potential of using 2D NMR in metabolomics. For instance, Van *et al.* showed that 2D TOCSY spectra of mice urine samples allowed to better characterize concentration changes in low-concentrated metabolites compared to classical 1D NMR (Van et al., 2008). Since then, 2D spectra have been used in a number of metabolomics studies that highlighted the relevance of using 2D NMR data in such context (Robinette et al., 2011; Féraud et al., 2015; Puig-Castellví et al., 2018). An additional benefit of 2D NMR is that it requires a less advanced level of pre-processing, since data are already well separated at the acquisition stage (Féraud et al., 2019).

**FIGURE 7:**
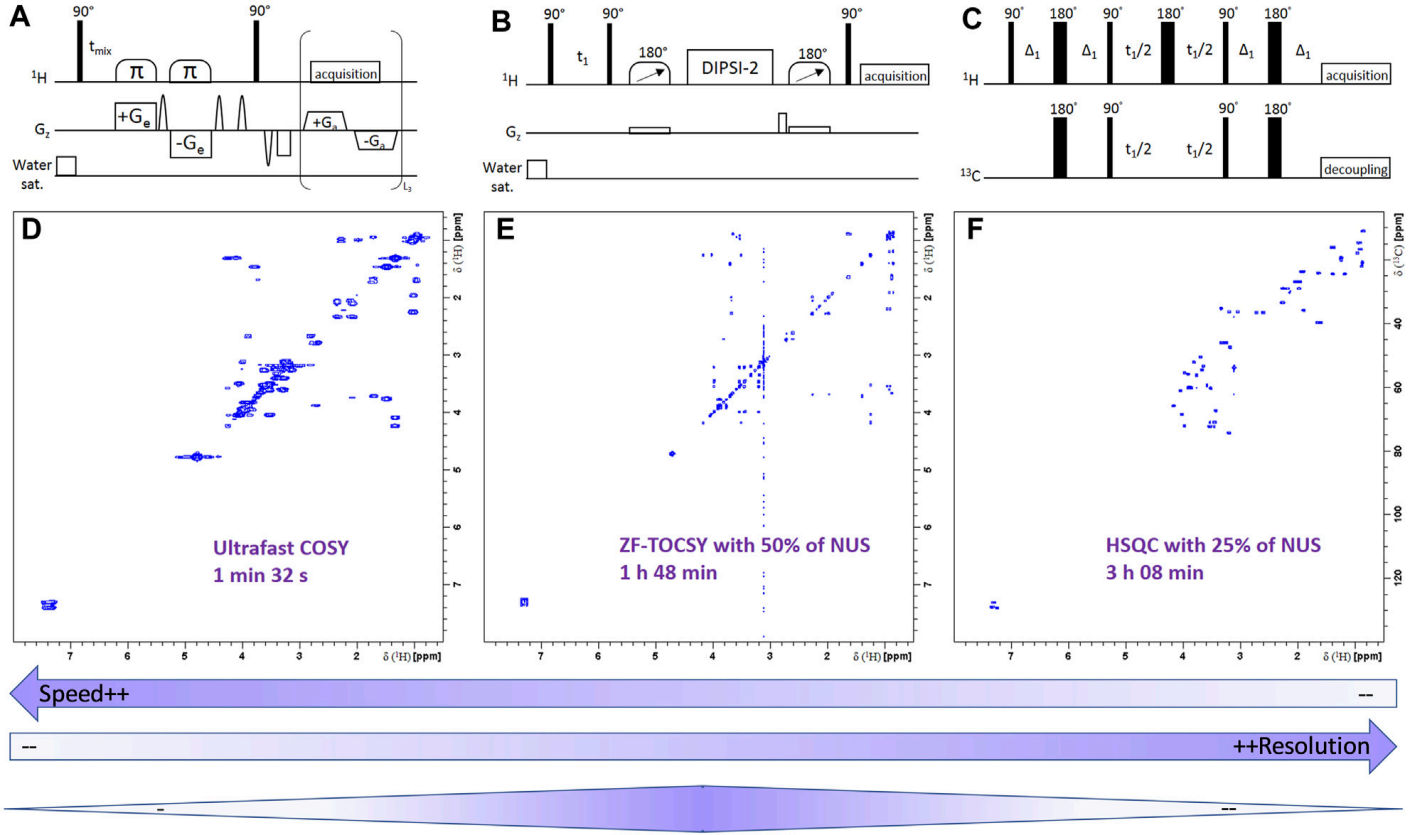
Typical fast 2D NMR pulse sequences for high-throughput metabolomics and their corresponding spectrum obtained on a model mixture of metabolites **(A,D)** Ultrafast ^1^H–^1^H correlation spectroscopy (UF COS)Y **(B,E)** Z-filter ^1^H–^1^H total correlation spectroscopy (ZF-TOCSY) with 50% of non-uniform sampling in the indirect dimension **(C,F)** ^1^H–^13^C heteronuclear single-quantum correlation (HSQC) with 25% of non-uniform sampling in the indirect dimension. Pulse sequences offer complementary performances in terms of speed, resolution and sensitivity. Adapted with permission from (Martineau et al., 2020).

However, a major obstacle which has limited the systematic use of 2D NMR in metabolomics has been the long experiment time needed to record 2D spectra with sufficient sensitivity and resolution. Indeed, typical 2D NMR experiments may last between a few tens of minutes until many hours since they rely on the repetition of numerous 1D experiments with a slight incrementation of a specific delay in the pulse sequence. Such durations are not compatible with the high-throughput analysis of large sample cohorts. They may also not be compatible with time stability issues of some biological samples. Fortunately, several methodological developments have been carried out to accelerate the acquisition of 2D NMR spectra (Rouger et al., 2016). These include spectral aliasing (Njock et al., 2010), fast repetition techniques (Schanda, 2009), non-uniform sampling (NUS) (Mobli and Hoch, 2014) or ultrafast (UF) spectroscopy (Giraudeau and Frydman, 2014). The reader is referred to the aforementioned reviews for detailed explanations on the corresponding methodologies, but in summary, these methods can accelerate the acquisition of 2D NMR spectra while preserving a good sensitivity and resolution performance, leading to reasonable acquisition times. A particularly interesting feature of fast 2D NMR is that it offers many different pulse sequences with complementary features that make it possible to choose, for a given matrix and application, the best compromise between experiment time, sensitivity and resolution (Figure 7) (Martineau et al., 2020). Some of these methods have been successfully applied in metabolomics workflows. For instance, Marchand *et al.* showed that UF COSY and NUS TOCSY applied to pig lipid serum lipid extracts offered an improved detection of biomarkers characterizing the administration of a growth promoted (Marchand et al., 2018). Feraud *et al.* showed that NUS COSY spectra recorded in less than 10 min provided a fast and efficient approach for the profiling of human urine samples (Féraud et al., 2020). It is also worth highlighting that most 2D NMR approaches only provide information on relative metabolite concentration variations between samples. However, when associated with appropriate analytical procedures, fast 2D NMR can yield accurate absolute quantitative data, which can be an interesting alternative to 1D NMR when targeted quantification of metabolites (Marchand et al., 2017). This strategy has been applied, for instance, to the quantification of metabolites in cancer cell extracts (Martineau et al., 2011; Le Guennec et al., 2012).

Another promising perspective on the pulse sequence development side arises from pure-shift NMR methods. The term “pure-shift” refers to an ensemble of methodologies that aim at transforming all NMR multiplets into singlets (Castañar, 2017). In the case of ^1^H NMR, pure-shift NMR provides a great way of reducing peak overlap in the case of complex mixtures, while retaining the simplicity of 1D spectra. These methodologies suffer from a strong sensitivity penalty but could be attractive for metabolomics workflows. Recent studies demonstrated the potential of pure-shift NMR in plant metabolomics (Lopez et al., 2019), and application to samples of clinical relevance may occur in the coming years.

Finally, an alternative to these pulse sequence approaches to simplify NMR spectra of complex mixtures is to rely on selective methods that reduce the number of observable analytes. While this strategy may seem counter-intuitive in metabolomics, such methods can be of interest when focusing on a limited set of targeted metabolites which can be of interest as markers of a given pathology. Most promising strategies rely on “chemosensing” methods such as the addition of charged nanoparticles that selectively suppress NMR signals of metabolites whose charge is opposite to those of the nanoparticles (Zhang et al., 2016), or the coating of nanoparticles with ligands that selectively bind to some classes of metabolites (Salvia et al., 2015).

### Improving the Accessibility

**FIGURE 8:**
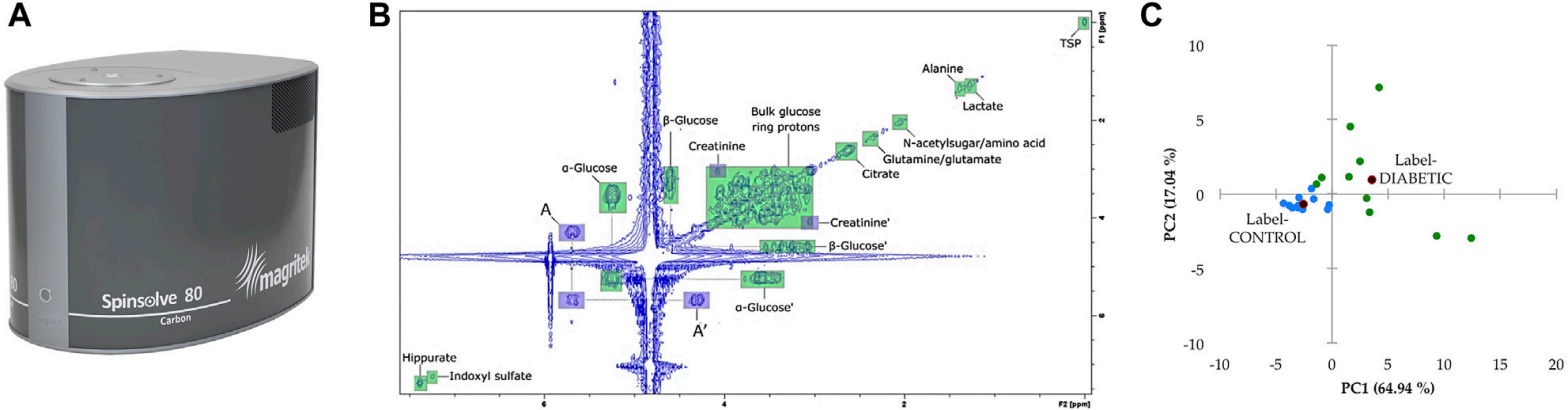
Potential of benchtop NMR spectroscopy to make NMR metabolomics more accessible in a clinical context. **(A)** Typical benchtop NMR spectrometer **(B)** 2D ^1^H–^1^H COSY NMR of type 2 diabetes urinary profile acquired at 60 MHz using a benchtop NMR spectrometer. Creatinine blue squares represent the long-range connectivity cross-peak for this metabolite. Blue squares labelled A represent unassigned, unusual doublet resonances arising from ‘mirroring’ spectral signal located at δ = 5.13–5.29 ppm (in this case, reflecting the α-glucose cross-peak). **(C)** Principal component analysis (PCA) scores plot of PC2 (17.04% of total variance) versus PC1 (64.94% of total variance) for a preliminary investigation of distinctions between healthy control and type 2 diabetic cohorts, and also potential sample outliers. Colour codings: blue, urine samples collected from healthy controls; green, those from type 2 diabetes participants. The black points represent scores plot centroids for the two groups explored. PCA was performed using XLSTAT2014 software, and the dataset was TSP-normalised, generalised logarithmically (glog)-transformed and Pareto-scaled prior to analysis. **(A)** Courtesy of Magritek GmbH. **(B)** Reproduced from (Leenders et al., 2020) under Creative Commons Attribution 4.0 International License. **(C)** Reproduced from (Percival et al., 2018) under Creative Commons Attribution 4.0 International License.

A major challenge for a widespread clinical application of NMR spectroscopy lies in the limited accessibility to NMR instruments arising from their high cost, heaviness and high level of technicity -including the regular handling of cryogenic fluids. Very exciting perspectives arise along this direction from the recent development of compact NMR spectrometers, which have been made commercially available for a few years and have already known a great success in chemistry labs and industries (Singh and Blümich, 2016). Such spectrometers rely on permanent magnets that do not require any specific maintenance and which provide a medium magnetic field (1–2 T) yielding a ^1^H resonance frequency between 40 and 100 MHz (Figure 8A) (Kuster et al., 2011). Benchtop NMR spectrometers are transportable (<100 kg), low-cost (<100,000 €) and most commercial models can easily be used in both static and flow configurations, which explains their success for chemical applications. Of course, they also have a reduced performance compared to high-field NMR spectrometer, with a lower sensitivity and a limited ability to separate overlapping peaks (owing to the small frequency range in Hz, while multiplet structures such as J-couplings are invariant to the magnetic field).

In this context, it goes without saying that benchtop NMR will not replace high-field NMR in the detection and structure elucidation of low-concentrated biomarkers. But it could play a significant role as an affordable and high-throughput metabolic profiling tool at the point-of-care, especially when sample amounts are not limited, *e.g.* urine samples. Along this line, impressive results have been achieved by Wilson and co-workers, who demonstrated that a 60 MHz commercial benchtop spectrometer could detect and even quantify major metabolites in urine within a few minutes, reaching limits of detection of *ca*. 25 µM (Percival et al., 2018). They also showed nicely resolved 2D COSY spectra (Figure 8B) (Leenders et al., 2020), and they eventually reported an efficient group separation between type 2 diabetes patients and healthy controls (Figure 8C). In another recent study, Izquierdo-Garcia *et al.* showed that a tuberculosis biomarker in urine -previously determined by high-field NMR-could also be detected by benchtop NMR (Izquierdo-Garcia et al., 2020).

While these results remain preliminary and will need to be validated at a larger scale, they highlight how NMR spectroscopy could soon make its way towards the patient’s bed and help as a routine tool for rapid and accurate sample classification in a clinical context. In addition, recent developments have shown how high-resolution pulse sequences could be implemented on benchtop spectrometers (Gouilleux et al., 2020). These include solvent-suppression pulse sequences such as those used in routine high-field NMR metabolomics, as well as methods which have been described above to improve the resolution such as fast 2D NMR or pure-shift approaches. Recent results highlighted the potential of such advanced benchtop NMR methods for sample classification (Gouilleux et al., 2018), and one can expect that clinical metabolomics will benefit from such advances in the coming years.

## Conclusion

Thanks to intrinsic properties such as high reproducibility, the possibility to quantify, a high degree of structural information, its “universal” detection capacity for all organic molecules as well as its adaptability in the analysis of biological samples, NMR very early appeared as a platform of choice in clinicals metabolomics. The numerous publications, works and results based on NMR attest this fact and keep contributing to the development of this approach. The recent progresses in mass spectrometry coupled with liquid or gas chromatography, a more sensitive and higher resolution technique, have progressively led NMR to play a “second” role in metabolomic studies, raising the question of its future in the field. However, the applications of metabolomics in clinical research and personalized medicine have brought new needs and challenges for metabolomics, such as the analysis of large cohorts, the stratification and the longitudinal follow-up of patients and the identification and quantification of biomarkers. To face such multiple requirements and needs, NMR has real assets and opportunities. Indeed, the many recent instrumental and methodological developments aiming at improving both sensitivity and resolution, as well as the demonstration of its excellent complementarity with mass spectrometry, highlight the leading role of NMR spectroscopy in clinical metabolomics and in personalized medicine. The translation from laboratory studies to clinical practice is another challenge that metabolomics is facing and, in this respect, we are confident that NMR will be one of the key analytical platforms that can provide valuable and innovative solutions and opportunities with a view to a personalized approach to medicine. Considering the numerous promising perspectives mentioned in this review, there is no doubt that the recent and future developments will rekindle the flame of NMR spectroscopy in clinical metabolomics for the next decades.
